# Cardiorespiratory Fitness and Motor Skills in Relation to Cognition and Academic Performance in Children – A Review

**DOI:** 10.2478/hukin-2013-0006

**Published:** 2013-03-28

**Authors:** Eero A. Haapala

**Affiliations:** 1 Department of Physiology, Institute of Biomedicine, University of Eastern Finland, Campus of Kuopio, Finland.

**Keywords:** physical fitness, movement skills, physical activity, children, scholastic achievement

## Abstract

Different elements of physical fitness in children have shown a declining trend during the past few decades. Cardiorespiratory fitness and motor skills have been associated with cognition, but the magnitude of this association remains unknown. The purpose of this review is to provide an overview of the relationship of cardiorespiratory fitness and motor skills with cognitive functions and academic performance in children up to 13 years of age. Cross-sectional studies suggest that children with higher cardiorespiratory fitness have more efficient cognitive processing at the neuroelectric level, as well as larger hippocampal and basal ganglia volumes, compared to children with lower cardiorespiratory fitness. Higher cardiorespiratory fitness has been associated with better inhibitory control in tasks requiring rigorous attention allocation. Better motor skills have been related to more efficient cognitive functions including inhibitory control and working memory. Higher cardiorespiratory fitness and better motor skills have also been associated with better academic performance. Furthermore, none of the studies on cardiorespiratory fitness have revealed independent associations with cognitive functions by controlling for motor skills. Studies concerning the relationship between motor skills and cognitive functions also did not consider cardiorespiratory fitness in the analyses. The results of this review suggest that high levels of cardiorespiratory fitness and motor skills may be beneficial for cognitive development and academic performance but the evidence relies mainly on cross-sectional studies.

## Introduction

Fewer than 20% of children meet the recommendations for health enhancing physical activity: at least 60 minutes of moderate to vigorous intensity physical activity, preferably on every day of the week (Verloigne et al., 2012). Furthermore, a decreasing trend in cardiorespiratory fitness has been documented (Tomkinson and Olds, 2007). Evidence indicates a similar trend in mastery of fundamental motor skills (Hardy et al., 2011).

Concerns have arisen as to whether today’s lifestyle has a similarly negative effect on children’s metabolic, cardiovascular, and brain health as it has on adults (Hillman et al., 2008). Physical activity has been found to be a foundation of development and reinforcement of motor skills (Riethmuller et al., 2009). Moreover, children with higher physical activity level also have a higher level of cardiorespiratory fitness, although the effect of physical exercise on cardiorespiratory fitness is smaller in children than in adults (Rowland, 2007). In addition, a higher level of physical activity has been related to better academic performance and executive functions in children and adolescents (Hillman et al., 2008). Executive function is an umbrella term for goal-oriented mental processes, which include planning, organising, behavioural inhibition, and working memory processes.

It is hypothesised that a positive effect of physical activity on cognitive functions is partly caused by physiological changes in the body such as increased levels of brain-derived neurotrophic factor (BDNF), that facilitates learning and maintains cognitive functions by improving synaptic plasticity and acting as a neuroprotective agent, increased brain circulation and improved neuroelectric functionality (Hillman et al., 2008). In animal studies it has been shown that different kinds of activities cause different adaptations in the brain. Endurance exercise is shown to increase capillary density (angiogenesis) and thereby to increase the brain blood flow whereas motor exercises are shown to cause synaptogenesis or an increased number of synapses (Adkins et al., 2006). Therefore, cardiorespiratory fitness and motor skills may be differently related to cognition and academic performance among children. Although higher physical activity level in general has been linked to improved cognitive performance, only few studies have compared aerobic and motor exercise in relation to cognitive functions among children and therefore, it is appropriate to compare cardiorespiratory fitness and motor skills although those measures are affected by maturation and genetic factors (Malina et al., 2004).

As children are becoming increasingly sedentary and unfit, it is important to recognise effective interventions to improve metabolic, cardiovascular, as well as cognitive health. The purpose of the present review is to summarise the evidence of associations of cardiorespiratory fitness and motor skills with some important components of cognition and brain functioning that are linked to academic performance among children up to 13-years of age. Also relevant literature of associations of cardiorespiratory fitness and motor skills with academic performance is described. These data is accompanied by the existence of physical or motor activity studies that report measures of cardiorespiratory fitness and motor skills. In addition, factors that may confound these results are discussed.

## Inhibitory control

Inhibitory control is the core of the higher cognitive functions called the executive control. Inhibitory control refers to higher order mental processes that are related to the control of attention, behaviour and emotions and involves mainly the neural networks in the prefrontal and parietal cortices (Diamond, 2013). Inhibitory control includes a selective attention to the requisite stimulus despite inappropriate or interfering stimuli and maintenance of information in working memory. Among children, inhibitory control is shown to be an important predictor of academic performance but also physical and mental health in adulthood and thereby, it is an important component of childhood cognitive development (Diamond, 2013).

The Eriksen flanker task is one of the most used tests for inhibitory control among children in fitness studies. The flanker task requires the child to identify as quickly and accurately as possible the direction of the centrally positioned arrow in either congruent (e.g. < < < < <) or incongruent (e.g. < < > > >) conditions. The demands of inhibitory control can be further increased using incompatible conditions. That is, the child is instructed to answer the opposite direction from the direction that the centrally presented arrow is pointing.

Evidence suggests that high levels of cardiorespiratory fitness is associated with a better response accuracy in the flanker task (Chaddock et al., 2012a; Chaddock et al., 2012b; Hillman et al., 2009; Pontifex et al., 2011; Voss et al., 2011) (Table 1). Moreover, some studies indicate that compared to unfit children, highly fit children are more accurate in the incongruent condition (but not in congruent condition) and have less variability in the response accuracy and reaction times in the flanker task conditions that involved a variable amount of interference (Pontifex et al., 2011; Voss et al., 2011).

Improved flanker task accuracy is accompanied by more efficient brain activation. Studies using event-related brain potentials (ERP) have shown that highly fit children have better inhibitory control and exhibit larger P3 amplitudes than unfit children (Hillman et al., 2009; Pontifex et al., 2011) (Table 1). The P3 is a positive-going component that is believed to reflect a subject’s attention to stimuli and processing speed. A larger P3 amplitude indicates an increased amount of attentional resources allocated towards a stimulus (Hillman et al., 2008). Moreover, highly fit children also have reduced error-related negativity (ERN) amplitude during the flanker task (Hillman et al., 2009). In addition, higher levels of fitness have been related to a larger change in ERN amplitude between the compatibility conditions (Pontifex et al., 2011).

The ERN is an ERP component that has been related to erroneous responses in tasks that emphasise reaction time. Some evidence indicates that the ERN is related to known mistakes and is therefore a marker for error detection and compensation, that is, action monitoring (Gehring et al., 1993). Furthermore, increased ERN may also be associated with an increased level of response conflict (Gehring and Fencsik, 2001) and reduced ERN may reflect more efficient action monitoring (Gehring et al., 1993). Thus, these results indicate that higher levels of cardiorespiratory fitness is related to a better attention allocation, action monitoring and increased modulation of cognitive control.

Cross-sectional studies using functional magnetic resonance imaging technique (fMRI) indicate that children with high levels of fitness exhibit more efficient neural activation and cognitive adaptation during the flanker task compared with less fit children (Chaddock et al., 2012b; Voss et al., 2011) (Table 1). Findings suggest that cortical activation does not differ between highly fit and unfit children during the congruent condition, but when cognitive demands increase during the incongruent condition, highly fit children maintain their response accuracy and exhibit greater activation in the prefrontal and parietal cortices in the early task period and reduced activation in the later periods indicating more effective cognitive flexibility (Chaddock et al., 2012b). Another study showed increased activation in the prefrontal cortex and decreased activation in the posterior parietal cortex of highly fit children during the flanker task (Voss et al., 2011). Moreover, highly fit children showed lower or the same activation than unfit children in the left central opecular cortex in the incongruent condition of the flanker task. Interestingly, higher activation was related to task performance only among highly fit children. Of these brain regions, the dorsolateral prefrontal cortex is involved in the manipulation of task relevant information within working memory (Diamond, 2013) and is related to top-down regulation of executive functions (MacDonald III et al., 2000). Also, the parietal cortex is involved in inhibitory control (Brass et al., 2005), and like the dorsolateral prefrontal cortex, parietal cortex is important for the working memory function (Hillman et al., 2008). Thus, the findings indicate that 9 to 10-year-old children with superior cardiorespiratory fitness are capable of activating their frontal and parietal circuitry in demanding cognitive tasks more efficiently than their less fit counterparts.

Moreover, some evidence indicates that better response accuracy among highly fit children in comparison with unfit children is related to larger subcortical structure volumes (Table 1). Chaddock et al. (2012a) found that compared to unfit children, highly fit children had larger bilateral putamen and glopus pallidus volumes and greater response accuracy during a modified flanker task in the baseline as well as in the one-year follow-up. Highly fit children also had shorter reaction times in the follow-up. The authors concluded that greater volumes of the putamen and the globus pallidus were associated with better cognitive control.

Also motor skills have been studied in relation to cognitive control. Among studies, the results are inconsistent and the magnitude of associations is dependent on the assessment of inhibitory control. Better manual dexterity (but not throwing accuracy or balance) has been associated with shorter reaction times in the modified day-night Stroop task whereas motor skills were not related to the reaction times during the Go/NoGo task (Livesey et al., 2006) (Table 1). Better whole body coordination (including sideways jumping speed and better ability to quickly change posture from lying down to an upright position and back to lying down i.e. postural flexibility) and better manual dexterity have been linked to shorter reaction times of correct responses during the Simon task (Roebers and Kauer, 2009). In addition, a better sideways jumping performance was associated with shorter reaction times in the flanker task, whereas other whole body coordination and manual dexterity skills were not related to the flanker task performance (Roebers and Kauer, 2009). However, these studies did not investigate relationships between motor skills and response accuracy. There is some vagueness whether reaction time is an appropriate measure of inhibitory control. Evidence suggests that children with high and low cardiorespiratory fitness did not differ in their reaction times in the flanker task but children with higher levels of fitness have better response accuracy than those with lower levels of fitness (Hillman et al., 2009; Pontifex et al., 2011).

## Working and short term memory

Working memory involves holding and manipulating the information in mind and relies on dorsolateral prefrontal cortex. Working memory involves also associative or relational memory. Relational memory involves learning about the relationship between two stimuli. In contrast, short term memory involves keeping information in mind without manipulating it, thus learning the properties of the stimulus (i.e. item memory). Short term memory does not need involvement of dorsolateral prefrontal cortex and it relies on ventrolateral prefrontal cortex (Diamond, 2013). In the behavioural point of view, working memory has been linked to academic performance among children. Moreover, inhibitory control is linked to working memory, thus inhibitory control is shown to support the working memory processes (Diamond, 2013).

The current evidence suggests that higher levels of cardiorespiratory fitness are related to a better relational memory and thereby hippocampal encoding (Chaddock et al., 2010; Monti et al., 2012) (Table 2). However, these studies did not find a relationship between cardiorespiratory fitness and item memory. In addition, a larger bilateral hippocampal volume has been related to a better relational memory and based on mediating analyses, bilateral hippocampal volume is shown to mediate the relationship between cardiorespiratory fitness and relational memory (Chaddock et al., 2010b) (Table 2). Moreover, Monti et al. (2012) extended these cross-sectional designs by conducting a longitudinal analyses to study whether nine-month aerobic training was related to working memory performance and eye movement patterns during a memory task in the follow-up. Children in the training group increased their VO_2max_ percentile whereas the VO_2max_ percentile among children in the control group slightly decreased. Children in the training group had a longer viewing time for displayed pictures during relational memory tasks that required the children to encode and correctly retrieve self-chosen sets of scenes and faces. In contrast, no differences in viewing time were observed during an item memory task that required retrieval of familiar faces without any need to relate them to a scene. Moreover, they found no differences in task accuracy or reaction times. Although no fitness differences were observed in response accuracy, these results indicated that increased cardiorespiratory fitness is related to more efficient hippocampal encoding. Evidence suggests that improved hippocampal encoding alone is not enough to elicit correct responses during relational memory tasks; high performance on tasks requires prefrontal cortex activation and efficient hippocampal prefrontal cortex connectivity (Hannula and Ranganath, 2009). Moreover, these results by Monti et al. (2012) should be interpreted cautiously because there was no baseline assessment for memory performance.

In addition, findings by Niederer et al. (2011) support other studies on a non-significant relationship between cardiorespiratory fitness and item memory (Table 2). However, Kamijo et al. (2011) conducted a RCT study whether aerobic exercise training over a nine-month period would have an effect on item memory and ERP component called contingent negative variation (CNV). The CNV reflects cognitive, motor, and sensory processes in preparation for an upcoming stimulus. The CNV includes at least two components, initial and terminal waves, which have different functions (Brunia and van Boxtel, 2001). The initial CNV wave has been associated with stimulus orientation (Weerts and Lang, 1973) whereas the late wave is mainly related to motor and response preparation (Brunia and van Boxtel, 2001). Kamijo et al. (2011) adopted a modified Stenberg task to assess a delayed recognition of previously presented arrays of letters. After the intervention, only the training group (relative to the control group) improved their VO_2max_ by 4.2 ml/kg/min, as well as their response accuracy in comparison to pre-intervention accuracy. Improved accuracy was observed only in the task condition, where the child was required to encode an array of three letters; no significant improvement was found when one or five letters were encoded. The exercise group also exhibited greater frontal initial CNV (iCNV) amplitude compared with controls on post-intervention testing. The authors speculated that cardiorespiratory fitness-related increases in iCNV amplitude indicate an increased efficiency of anticipatory attention and pronounced top-down control. However, these results concerning the effect of cardiorespiratory fitness on item memory are in contrast to other studies showing no relationship between cardiorespiratory fitness and item memory. Therefore, improved item memory may be due to physical exercise per se, without a mediating effect of cardiorespiratory fitness.

The relationship between motor skills and memory has been investigated using mainly item memory tests. Both, a better whole body coordination and manual dexterity, have been associated with a better item memory (Niederer et al., 2011; Piek et al., 2008; Roebers and Kauer, 2009; Wassenberg et al., 2005) (Table 2). Furthermore, some evidence indicates that earlier gross motor development predict item memory at later years (Piek et al., 2008). Moreover, motor skills may be differently related to working memory depending on whether the assessment of motor skills is an outcome oriented (e.g. distance in the standing long jump) or a process oriented (e.g. throwing technique). Wassenberg et al. (2005) demonstrated no relationship between motor test outcomes, but showed a positive relationship between fluency and technique during the motor task and item working memory test performance. Moreover, in one study, more agile children showed superior item recall than their less agile peers. Furthermore, children with better dynamic balance at the baseline showed significant improvement in item memory during a nine-month follow-up (Niederer et al., 2011). However, not all studies have found a relationship between motor skills (manual dexterity) and spatial working memory (Pangelinan et al., 2011) indicating some specificity of effects of cardiorespiratory fitness and motor skills on memory functions.

## Academic performance

Academic performance refers to a child′s success in school, measured by grade point averages or the child’s meeting standards of various achievement tests. In addition to plain academic knowledge, grade point averages usually contain information about class attendance or classroom behaviour, whereas standardised tests rely on academic knowledge. Academic performance is affected by inhibitory control and working memory performance, as well as efficiency of other aspects of executive control (Diamond, 2013).

Cross-sectional studies suggest that higher cardiorespiratory fitness is related to better results and higher scores in standardised achievement tests (Van Dusen et al., 2011; Welk et al., 2010; Wittberg et al., 2010, Davis and Cooper, 2011) and to higher scholastic ratings (Dwyer et al., 2001) in comparison to peers with a lower fitness level (Table 3).

Most studies have addressed the relationship between cardiorespiratory fitness and academic performance using field-based exercise tests and cut-offs for cardiorespiratory fitness (Van Dusen et al., 2011; Welk et al., 2010). These studies indicate that achieving a certain cardiorespiratory fitness level is significantly and positively associated with improved academic performance (Table 3). Studies that have adopted linear methods also indicate that higher cardiorespiratory fitness is related to better academic performance (Dwyer et al., 2001; Wittberg et al., 2010; Davis and Cooper, 2011). Furthermore, these studies suggest that the degree of the association between cardiorespiratory fitness and academic performance depends on the assessment of cardiorespiratory fitness. Whereas Dwyer et al. (2001) did not find a significant association between academic performance and cardiorespiratory fitness estimated by a cycle ergometer, a significant association was found when they used time spent on a 1.6 km running test as a measure of cardiorespiratory fitness. Instead, one study found that low running time in one mile test was associated with better academic performance, whereas no association was found between endurance shuttle run test and academic performance among 9- to 13-year-old boys. In contrast, the association between cardiorespiratory fitness and academic performance was only found with endurance shuttle run performance in girls (Wittberg et al., 2010) (Table 3).

In contrast to studies concerning the relationships of cardiorespiratory fitness to academic performance that have used grade point averages and standardised achievement tests as measures of academic performance, associations of motor skills with academic performance include measures of grade point averages, estimated academic performance by a class-room teacher and reading skills. The evidence indicates that children with better overall perceptual motor abilities have better grade point averages than children with lower perceptual motor skills. Positive associations have also been found between manual coordination, static balance, asynchronous movement, and grade point averages in girls who were 10–11 years of age (Nourbakhsh, 2006).

Evidence from longitudinal studies indicates that only fine motor skills in young childhood predict improvement in reading and math skills in later years (Pagani et al., 2010). However, none of the studies involved an analysis of the possible relationship between current motor skills, cognition, and academic performance.

Intervention studies aimed at improving motor skills have documented a weak positive connection between motor skill training and improved academic performance and reading skills among school-aged children (Ericsson, 2008; Uhrich and Swalm, 2007), but the evidence is not consistent (Bassin and Breihan, 1978). Ulrich and Swalm (2007) showed that children who participated in bimanual dexterity training for six weeks improved their reading comprehension, but not reading decoding in comparison with the children in the control group.

Furthermore, Ericsson (2008) showed that children who were assigned to a physical training programme for motor skills not only improved their motor skills, but also their Swedish language (the first language of the participants) and mathematic performance. However, Bassin and Breihan (1978) documented no effect of twenty-week visuomotor training on reading achievement.

## Discussion

The findings presented in this review suggest that both higher levels of cardiorespiratory fitness and better motor skills are linked to a higher cognitive capacity and a better academic performance. However, cardiorespiratory fitness and motor skills may be differentially related to cognitive functions. Higher levels of cardiorespiratory fitness are mainly associated with a higher performance in tasks that require rigorous attention allocation and the use of attention resources in order to prevent undesirable results (Buck et al., 2008; Chaddock et al., 2010a; 2011; 2012; 2012a; 2012b; Hillman et al., 2005; 2009; Niederer et al., 2011; Pontifex et al., 2011; 2012; Voss et al., 2011; Wu et al., 2011) and a higher performance in the memory tests that involves hippocampal encoding (Chaddock et al., 2010b; 2011; Monti et al., 2012). In contrast, it seems that higher levels of cardiorespiratory fitness do not affect the performance on item memory tests. Furthermore, children with high levels of fitness have a better academic test performance than those with lower levels of fitness (Castelli et al., 2007; Davis and Cooper, 2011; Dwyer et al., 2001; Edwards et al., 2011; Eveland-Sayers et al., 2009; Roberts et al., 2010; Van Dusen et al., 2011; Welk et al., 2010; Wittberg et al., 2010).

Better motor skills have been related to a better performance in various cognitive tests including tasks for IQ, attention, inhibitory control, item memory and academic performance (Livesey et al., 2006; Niederer et al., 2011; Nourbakhsh, 2006; Pangelinan et al., 2011; Piek et al., 2008; Roebers and Kauer, 2009; Wassenberg et al., 2005). Furthermore, evidence indicates that early fine motor skills predict better academic performance later in life (Grissmer et al., 2010; Pagani et al., 2010). However, there may be an overlap between cross-sectional and cohort studies. If this is the case, children who develop earlier may also be more skilful in later childhood. Although it is inconsistent, some existing evidence indicates that motor skill training can contribute to improved academic skills and academic performance in children (Ericsson, 2008; Uhrich and Swalm, 2007). Furthermore, only one study has assessed both cardiorespiratory fitness and motor skills, but they were used in separate analyses (Niederer et al., 2011); thus, none of the studies examining this aspect controlled their analyses for cardiorespiratory fitness although it is known that cardiorespiratory fitness is related to motor skills (Lubans et al., 2010).

Regardless of relatedness of cardiorespiratory fitness and motor skills, results summarised in the present review emphasise that children should engage in various physical activities including both quantitative and qualitative aspects to contribute to cognitive development. That is, physical activity should incorporate with activities improving cardiorespiratory fitness and activities that enhance motor skills. However, this conclusion is mainly drawn from cross-sectional studies and, therefore, it cannot be concluded that exercise training to increase cardiorespiratory fitness and motor skills actually contributes to enhanced cognitive performance in children. In addition, this mainly cross-sectional data may be confounded by genetic factors, maturity status and other factors.

## Interplay of cardiorespiratory fitness, motor skills, genetics, and maturation

Although cardiorespiratory fitness and motor skills evidently play a role in cognition and academic performance, the extent to which these differences are mediated by genetic or biological factors is unclear. It is known that genetic factors can explain approximately 50% of cardiorespiratory fitness (Malina et al., 2004) and moreover, many childhood cognitive deficits are highly inheritable (Rutter, 2006). Cognitive deficits, such as learning disabilities, are often associated with coordination and motor skill disorders. Some authors have suggested that coordination disorders and learning disabilities may share a genetic cause (Martin et al., 2006). Again, there is some evidence that genetically regulated biological maturation may affect the relationships presented in this review. Boys who mature early show better motor skills than average and late maturing boys during early adolescence (Malina et al., 2004). However, the association between maturity and motor skills is somewhat mixed in girls (Malina et al., 2004). These results indicate that, at least in boys, biological maturity may confound the relationship between motor skills and cognitive functions. This means that more mature children may have an advanced neuromuscular system, leading them to perform better in motor tests. Moreover, children with older skeletal age, a marker of biological maturity, show better cognitive capacity in comparison to children with younger skeletal age (Goldstein, 1987). Thus, the relationship between motor skills and cognitive functions may be at least partly explained by differences in genetic background and maturation status. In contrast, the majority of studies that addressed relationships between cardiorespiratory fitness and cognitive functioning controlled their data for sexual maturity; that is, children who participated in those studies were classified as prepubertal according to their secondary sex characteristics (Chaddock et al., 2010; 2012a; 2012b; Hillman et al., 2009; Monti et al., 2012; Pontifex et al., 2011; Voss et al., 2011). In those studies, it is less likely that maturation confounded the results. Moreover, although genetic factors may have had an influence on the results presented here, genes usually act in a probabilistic fashion; thus, environment and other factors can downregulate or upregulate gene expression (Rutter, 2006). In this respect, the intervention results (Ericsson, 2008; Kamijo et al., 2011; Uhrich and Swalm, 2007) are encouraging in showing that training which improves cardiorespiratory fitness and/or motor skills may be beneficial for brain function and learning in childhood.

## Interplay between cardiorespiratory fitness and motor skills

It is an oversimplification to suggest that only cardiorespiratory fitness or only motor skills are the important determinants of cognitive functions in children. In fact, the evidence suggests that cardiorespiratory fitness and motor skills are positively correlated, suggesting that children with high cardiorespiratory fitness also have better motor skills than those with low fitness (Lubans et al., 2010). In this respect, it is not known whether exercise training consisting of aerobic exercise (for example treadmill running) without any component of motor skill training improves cognitive functions. Although Kamijo et al. (2011) showed that improved cardiorespiratory fitness enhanced cognitive performance, their intervention also consisted of exercises that potentially improved motor skills and musculoskeletal fitness. Results by Ericsson (2008), in contrast, indicated that children who improved motor skills through increased motor skill training improved their academic performance as well. However, it is likely that children who underwent physical activity intervention also saw an improvement in cardiorespiratory fitness. Thus, although aerobic walking has been related to improved cognitive functions in older adults (Hillman et al., 2008), it remains unclear as to whether simple aerobic exercise (such as walking) or motor skill training without an aerobic component, assists developing cognitive functions and brain health in growing children. On the other hand, it may be the case that aerobic training combined with motor skill training produces the best combination for cognitive development. Exercise training that leads to improved cardiorespiratory fitness creates an environment that supports neural growth and survival via increased levels of nerve growth factors, and motor skill training increases and refines structural efficiency of neural networks in children (Adkins et al., 2006).

## Situating the results into the context of the obesity epidemic

In the point of view of public health, the findings reviewed here are of great importance. The number of obese children continues to increase (Janssen et al., 2005), and a growing amount of children and adolescents spend their waking hours in sedentary activities (Verloigne et al., 2012). Moreover, a recent study showed that excess adiposity and especially intra-abdominal adiposity assessed by dual-energy X-ray absorptiometry (DXA) was negatively associated with inhibitory control and academic performance in 7- to 9-year-old children (Kamijo et al., 2012). Studies suggest that obese children have lower cardiorespiratory fitness (Stigman et al., 2009) and poorer motor skills (D’Hondt et al., 2012) in comparison to their normal weight peers. Moreover, more physical activity leads to decreased adiposity and improved motor skills in young children 0 to 4 years of age (Timmons et al., 2012). This negative relationship between adiposity and cognitive function may be partially explained by the poor development of cardiorespiratory fitness and motor skills through a lack of physically and mentally challenging activities. Moreover, there exists a threat that as younger children increasingly become sedentary and obese, they do not reach the level of physical fitness (including cardiorespiratory and motor components) necessary to support brain health and development. However, physical activity consisting of moderate to vigorous aerobic exercise and demanding motor skill exercises seems to be an effective intervention to enhance cognitive functioning, academic performance and brain health among overweight children (Davis et al., 2011b). The children in the training group, relative to the control group, showed increased activation in the bilateral prefrontal cortex and decreased activation in the bilateral posterior parietal cortex during the antisaccade task (Davis et al., 2011b) indicating more efficient cognitive processes. Unfortunately, the authors did not report task performance in relation to brain activation. Thus, there is some evidence that interventions aimed at increasing children’s physical activity levels and improving cardiovascular and neuromuscular health also have the potential to contribute to cognitive development.

## Conclusion

The evidence summarised in the present review suggests that compared to less fit children, highly fit children have larger subcortical brain structures, more efficient brain activation and neuroelectric efficiency during cognitive tasks, superior inhibitory control, working memory, attention and better academic performance. Similarly, children with better motor skills show better inhibitory control, attention capacity and academic performance than children with poorer motor skills. Thus, the evidence suggests that both cardiorespiratory fitness and motor skills play an important role in cognitive development during childhood. It is strongly suggested that children are allowed to get appropriate stimulation from the environment via physical activities in order to improve the functions of their cardiovascular and neuromuscular systems. Besides the known cardiometabolic benefits, developmentally appropriate physical activity may improve brain health and function. There is a need for multidisciplinary studies that assess the interdependent relationships of cardiorespiratory fitness and motor skill with cognitive functions in cross-sectional and longitudinal settings.

## Figures and Tables

**Table 1:** Summary of relationship of cardiorespiratory fitness and motor skills with inhibitory control

**Reference, country**	**Design/subjects**	**Fitness or motor skill**	**Inhibitory control**	**Main results**
Chaddock et al. (2012a), USA	Cross-sectional / semi-prospective 32 children aged 9–10 years	Maximal treadmill test^a^	Modified flanker task + MRI	HF had better accuracy in congruent and incongruent conditions at baseline than UF. After follow-up, HF showed superior performance in accuracy and RT showing better cognitive control. **MRI**. HF had larger bilateral putamen and GP volumes and these volumes correlated with response accuracy during incompatible condition. Putamen volumes were directly related to follow-up accuracy and RT in incompatible and compatible conditions
Chaddock et al. (2012b), USA	Cross-sectional 32 children 9–10 years of age	Maximal treadmill test^a^	Modified flanker task + fMRI	No differences in congruent task in activation levels between UF and HF. As cognitive demands increased in the incongruent, relative to congruent, condition, HF showed greater activation in the prefrontal and parietal cortex during early task block. Only HF showed a decrease in these brain areas and maintenance of accuracy
Hillman et al. (2009), USA	Cross-sectional 38 children 9 years of age	Endurance shuttle run test^b^	Modified flanker task + ERP	HF were more accurate across task conditions than UF. Greater P3 amplitude and reduced ERN was observed in HF than UF.
Livesey et al. (2006), Australia	Cross-sectional 36 children 5–6 years of age	MABC	Go/NoGo task, day-night stroop task	Better fine motor skills were associated with shorter reaction times in the Stroop task, but no association with Go/NoGo task performance was found
Pontifex et al. (2011), USA	Cross-sectional 48 children 10 years of age	Maximal treadmill test ^a^	Modified flanker task + ERP	HF showed greater response accuracy in compatible and incompatible condition than UF. Difference was larger in incompatible condition indicating that the harder the task, the larger the difference. HF demonstrated a larger change in P3 amplitude and ERN between compatibility conditions than UF
Roebers and Kauer (2009), Switzerland	Cross-sectional 112 children 7.5 years of age	Jumping moving sideways, postural flexibility, pegboard	Flanker task, Simon Task, Cognitive flexibility task (RT)	Pegboard test performance was directly associated with reaction time in Simon and Cognitive flexibility tasks. Performance in the postural flexibility test was directly related to reaction times in Simon task. Performance in the sideways moving task was directly associated with reaction times in Cognitive flexibility task. Jumping was directly associated with reaction times during flanker, Simon, and Cognitive flexibility tasks
Voss et al. (2011), USA	Cross-sectional 28 children 9–10 years of age (25 young adults 18–30 years served as a control)	Maximal treadmill test^a^	Flanker task + fMRI	HF exhibited greater overall accuracy percentage and greater accuracy in incongruent condition than UF. HF also showed less variation between congruent and incongruent condition than UF. ***fMRI.*** Children showed poorer activation than young adults. HF demonstrated more activation of brain regions involved in cinculo-opercular network

HF, Highly fit children. UF, unfit children. fMRI, functional magnetic resonance imaging.

MRI, magnetic resonance imaging. GP, glopus pallidus. ERP, event-related brain potential.

RT, reaction time. ERN, error-related negativity.

a *Divided into > 70^th^ and < 30^th^ percentile according to child’s VO_2max_*.

**Table 2:** Summary of relationship of cardiorespiratory fitness and motor skills with memory functions

**Reference, country**	**Design/subjects**	**Fitness or motor skill**	**Memory**	**Main results**
Chaddock et al. (2010), USA	Cross-sectional 59 children 9–10 years of age	Maximal treadmill test^a^	Item and relational memory paradigm + MRI	HF had greater bilateral hippocampal volumes and better relational memory than UF; bilateral hippocampal volume mediated the relationship between fitness and relational memory. No association between fitness and item memory was found
Kamijo et al. (2011), USA	RCT (9 months) 36 children 7–9 years of age	Maximal treadmill exercise test to assess VO_2max_ before and after exercise intervention	Modified Stenberg task for item memory + ERP	Increased fitness after the intervention was associated with improved overall accuracy in memory task. The intervention group exhibited greater iCNV amplitude in comparison to control group at the end of the intervention period
Monti et al. (2012), USA	Longitudinal (9 months) 44 children 9 years of age, cognitive assessment of only in the follow-up	Maximal treadmill exercise test to assess VO_2max_ before and after exercise intervention	Working memory task for non-relational and relational memory, eye-movement tracking	Children in the exercise intervention group showed longer viewing times for the related pictures than control group during relational memory task. No differences during non-relational memory task accuracy. No differences in performance accuracy or reaction times between intervention and control groups
Niederer et al. (2011), Switzerland, Germany	Cross-sectional /longitudinal (nine-month follow-up) 245 children 5.2 years of age	Agility, dynamic balance, multistage shuttle run test	Item memory	Agility was associated with item memory in cross-sectional analyses and baseline balance predicted improvement in item memory longitudinally
Pangelinan et al.(2011), USA	Cross-sectional 172 children 6– 13 years of age	Purdue pegboard task	Spatial working memory + MRI	Pegboard test performance was not related to spatial working memory.
Piek et al. (2008), Australia	Cohort study 33 children 6–11 years of age at the follow-up (initial assessment at the age of four month)	Ages and stages questionnaire, McCarron assessment of neuromuscular development	Digit span and letter-number sequencing = working memory index	Early gross motor development predicted working memory
Roebers and Kauer (2009), Switzerland	Cross-sectional 112 children 7.5 years of age	Sideway jumping, moving sideways, postural flexibility, pegboard	Backward colour recall	Better performance in the postural flexibility test was related to higher performance in memory task
Wassenberg et al.(2005), the Netherlands	Cross-sectional 378 children 6.18 years of age	Maastricht motor test (static balance, dynamic balance, ball skills, manual dexterity)	Auditory item memory	Better qualitative motor skills (i.e. the movement process) were related to a better item memory

HF, highly fit children. UF, unfit children. ERP, event-related brain potential.

iCNV, initial contingency negative variation

a Divided into > 70^th^ and < 30^th^ percentile according to child’s VO_2max_.

**Table 3:** Summary of relationship of cardiorespiratory fitness and motor skills with academic performance

**Reference, country**	**Design/subjects**	**Fitness or motor skills assessment**	**Academic performance assessment**	**Main results**
Bassin and Breihan (1978), USA	Intervention 66 children	Visuomotor activities for trice a week for 20 weeks	Reading achievement	Motor skill based intervention had no effect on reading achievement.
Davis and Cooper (2011), USA	Cross-sectional 170 overweight children 7–11 years	Maximal treadmill test	The Woodcock-Johnson III	Higher levels of cardiorespiratory fitness was associated with better reading and arithmetic performance
Dwyer et al. (2001), Australia	Cross-sectional 7961 children 7–15 years of age	1.6 km, indirect submaximal cycle ergometer exercise test	Scholastic ability rated by 5-point scale	Better performance in the 1.6 km run was associated with higher scholastic ability
Ericsson (2008), Sweden	Intervention study 251 primary school children in Grades 1 to 3	Motor activities for five lessons / week	Swedish (writing and reading), mathematics	Significant association between participation to the intervention and academic performance
Nourbakhsh (2006) Iran	Cross-sectional 400 girls 10–11 years of age	The Oseretsky scale	Grade point average	General static and dynamic coordination, synchronous-symmetrical and asynchronous-asymmetrical movements and total perceptual motor skill correlated positively with grade point average
Pagani et al. (2010), Canada	Cohort study 1145 children (538 boys) 5.4 years of age	Gross motor skills, fine motor skills	Teachers’ estimation of academic performance	Fine motor skills at the age of five predicted mathematics, reading, and overall academic performance 24 months later
van Dusen et al. (2011), USA	Cross-sectional 254 743 third to 11th grade students	1 mile run, endurance shuttle run test (lowest quintile vs. highest quintile)	Texas Assessment of Knowledge and Skills (reading and mathematics)	High-fit children had better academic performance, but no differences was found in elementary school
Uhrich and Swalm (2007), USA	Cluster-RCT 41 children 8 years of age	Speed stacking	Gates-MacGinitie Reading Test (decoding, comprehension)	Intervention group performed better in comprehension but no differences were observed in decoding
Welk et al. (2010), USA	Cross-sectional 36 835 students	Endurance shuttle run test, 1.6 km run (meets recommendations vs. does not meet recommendations),	Texas Assessment of Knowledge and Skills; attendance, delinquency	Meeting recommendations for aerobic fitness was associated with better academic performance and better attendance
Wittberg et al. (2010), USA	Cross-sectional 1740 children 9–13 years of age	1 mile run, endurance shuttle run test. (time spent to complete 1 mile; number of circuits in the shuttle run test)	West Virginia Standardized Test	Higher number of circuits in the PACER was associated with better academic performance in girls and faster time in the 1 mile run was associated with higher academic performance in boys
